# Comparison of short- and long-axis nerve hydrodissection for carpal tunnel syndrome: A prospective randomized, single-blind trial

**DOI:** 10.7150/ijms.63815

**Published:** 2021-08-13

**Authors:** Si-Ru Chen, Tsung-Yen Ho, Yu-Ping Shen, Tsung-Ying Li, Yu-Chi Su, King Hei Stanley Lam, Liang-Cheng Chen, Yung-Tsan Wu

**Affiliations:** 1 Department of Physical Medicine and Rehabilitation, Tri-Service General Hospital, School of Medicine, National Defense Medical Center, No. 325, Sec. 2, Cheng-Kung Road, Neihu District, Taipei, Taiwan, Republic of China.; 2 Department of Physical Medicine and Rehabilitation, Taichung Armed Forces General Hospital, No. 348, Sec. 2, Chungshan Road, Taiping District, Taichung City, Taiwan, Republic of China.; 3 Integrated Pain Management Center, Tri-Service General Hospital, School of Medicine, National Defense Medical Center, No. 325, Sec. 2, Cheng-Kung Road, Neihu District, Taipei, Taiwan, Republic of China.; 4 The Hong Kong Institute of Musculoskeletal Medicine, Hong Kong.; 5 Department of Family Medicine, the Chinese University of Hong Kong, Hong Kong.; 6 Department of Family Medicine, the University of Hong Kong, Hong Kong.

**Keywords:** Carpal tunnel syndrome, hydrodissection, short-axis, long-axis

## Abstract

**Background:** This study is to compare the efficacy of short-axis hydrodissection with long-axis hydrodissection for patients with mild-to-moderate carpal tunnel syndrome (CTS).

**Methods:** Forty-seven patients with mild-to-moderate CTS were enrolled in a prospective, randomized, single-blinded, controlled trial (6 months follow-up). With ultrasound guidance, patients in both groups (short-axis or long-axis groups) were injected with normal saline (5 mL per session). Assessments were performed before and 2 weeks after the injection, as well as at 1, 3, and 6 months post-intervention. The primary outcome measure was the Boston Carpal Tunnel Syndrome Questionnaire (BCTQ) score and secondary outcomes included the cross-sectional area of the median nerve and electrophysiological studies.

**Results:** Forty-four patients (21 wrists in the short-axis group and 23 wrists in the long-axis group) completed the study. Compared with the baseline, both groups showed improved BCTQ and cross-sectional area at all follow-up assessments (p<0.05). The short-axis group was not more effective except significant improvements in BCTQ-severity and BCTQ-function 1 month post-injection compared to the long-axis group (p = 0.031 and p = 0.023, respectively).

**Conclusions:** Both short- and long-axis hydrodissection were effective for patients with mild-to-moderate CTS and the short-axis approach was not more effective than long-axis injection. Further studies with larger sample sizes, multiple injections, and larger injection volume are encouraged in the future.

## Introduction

Carpal tunnel syndrome (CTS) is the most prevalent peripheral compressive neuropathy 1. Although the exact pathophysiology remains nebulous, the prevailing theory is that progressive subsynovial tissue fibrosis and ischemia of the median nerve (MN) along with high pressure in the carpal tunnel 2, 3.

Nerve hydrodissection (HD), a method used to release adhesions by dissecting the anatomic spaces with fluid injection 4, was recently found to facilitate ultrasound-guided nerve injection for treating entrapment neuropathy 5-8. The therapeutic rationale is to detach nerve from surrounding connective tissue, increasing the blood flow, and allowing the nerve impulses to pass 5-8. Research published in 2019 demonstrated the efficacy of HD to treat mild-to-moderate CTS 9. Despite the positive clinical effects of HD for CTS, whether the injection technique would influence the duration of the HD effect is still unknown.

Currently, the two main methods of ultrasound-guided perineural injection for CTS, short- or long-axis approaches, are broadly applied in clinical practice 10-13. The method of long-axis injection may directly separate the MN from the flexor retinaculum (FR) with more contact area 14. In contrast to long-axis injection in which only the FR is hydrodissected from the MN, the operator can simultaneously hydrodissect the FR and subsynovial connective tissue (SSCT) through the short-axis approach. Furthermore, the short-axis approach could provide more accuracy with a lesser incidence of nerve injury because the operator can clearly identify the MN between the FR and SSCT with ultrasound guidance 15. However, due to inconsistent results from published studies, controversy continues about the optimal strategy between short- and long-axis injection for CTS 12, 13, 16-18. In our clinical practice, both short- and long-axis injections were effective for HD in patients with CTS. Moreover, we observed that hydrodissecting both the FR and the SSCT seemed to enhance the therapeutic benefits of HD, based on patients' clinical presentations. Thus, we hypothesized that short-axis HD may have equivalent or superior efficacy than long-axis HD for treating CTS. Hence, this trial aimed to compare short- and long-axis approaches in patients with mild-to-moderate CTS.

## Materials and Methods

### Study design

This prospective, randomized, single-blind controlled study was conducted with the approval of the institutional review board of our institute (No. 2-106-05-042) and was officially listed and accepted at www.ClinicalTrials.gov with the registration number NCT03031041. From January 2017 to August 2019, 50 individuals diagnosed with mild-to-moderate CTS were eligible, and 47 were enrolled in this study. Written informed consent for this trial was received from all the patients. An independent researcher utilized computer-generated randomization (Microsoft Excel) to determine which procedure the patient received. All subjects were block-randomized in a 1:1 ratio.

Both groups underwent one session ultrasound-guided HD with 5 mL of normal saline (NS). Both wrists were allocated to the same group assuming that participants were diagnosed with bilateral CTS and only the most symptomatic side was recorded for analysis. Except for acetaminophen (500 mg, up to 4 g per day), any treatment for CTS was prohibited for 2 weeks before and 6 months after the injection. A research assistant performed regular follow-ups on whether additional therapies were utilized.

### Inclusion and exclusion criteria

This study enrolled 20-80-year-old subjects with mild-to-moderate CTS authenticated by electrophysiological study and symptoms for at least 3 months. Symptoms and signs of CTS included: 1) positive Phalen's test or Tinel's sign, 2) decreased sensation with numbness over MN innervated territory of hand, 3) MN innervated thenar muscle weakness or atrophy, and 4) paresthesia, dysesthesia, or pain aggravated by long rest or repeated wrist motion, and relieved by shaking the hand or changing the posture. If patients met the first criterion, along with one or more of the remaining criteria, they were diagnosed with clinical CTS 6, 19. Exclusion criteria included: 1) a previous history of wrist surgery, polyneuropathy, brachial plexopathy, or thoracic outlet syndrome, 2) systematic infection, 3) pregnancy, or 4) previous steroid injection for CTS.

### Electrophysiological study and grades

The patients diagnosed with mild-to-moderate CTS based on the electrophysiological severity grade by Padua et al. 20-22: mild: normal distal motor latency (DML) with abnormal digit/wrist sensory nerve conduction velocity (SNCV); moderate: both digit/wrist SNCV and DML were abnormal; severe: SNCV was absent and DML was abnormal.

### Ultrasound-guided short- and long-axis injection (Figure 1)

**Figure 1:**
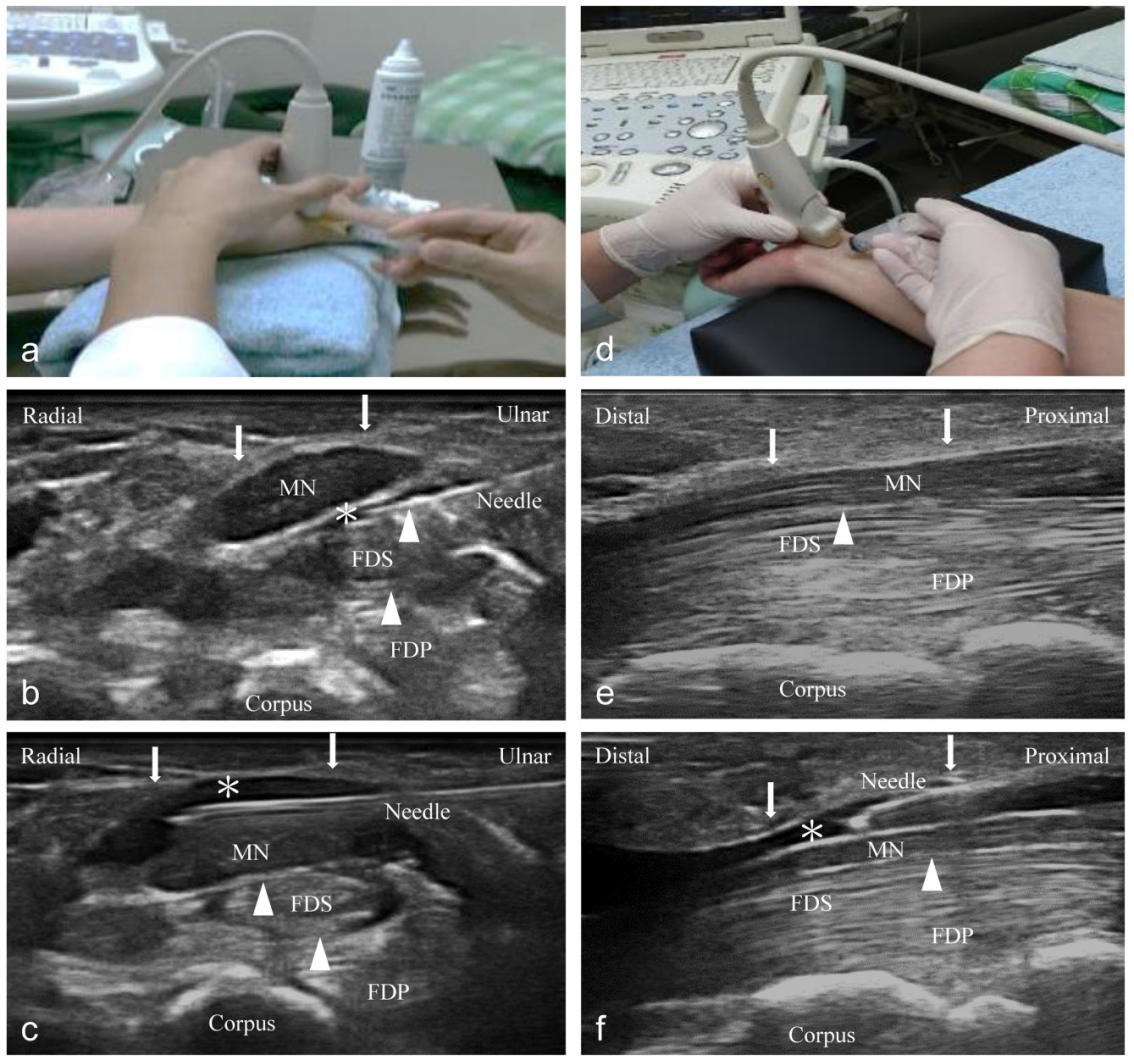
Ultrasound-guided injection image (left: short-axis injection; right: long-axis injection). **(a)** The position of in-plane short-axis injection at proximal inlet of the carpal tunnel. **(b)** The short-axis view shows that the MN separated from the subsynovial connective tissue (arrowheads) via hydrodissection (HD) (*: Injectate). **(c)** The short-axis view shows that the MN was separated from the flexor retinaculum (FR) (arrows) via HD (*). **(d)** The position of the in-plane long-axis injection advancing from the wrist crease to the palm. **(e)** The long-axis view shows swollen nerve fascicles, FR (arrows), and inflamed tendons in the same plane. **(f)** The long-axis view shows that the MN separated from the FR (arrows) via HD. MN: median nerve; FDS: flexor digitorum superficialis; FDP: flexor digitorum profundus.

An independent physician with 7 years of experience performed ultrasound-guided injections with a 10-18-MHz liner-array transducer (MyLab™25Gold, Esaote, Genova, Italy) 6. A 25-gauge, 2-inch needle was used, without the administration of a local anesthetic throughout the procedure. At the scaphoid-pisiform level, the MN was observed at the inlet of the carpal tunnel. In the short-axis group, we used 2 mL NS to hydrodissect the MN from the SSCT with an in-plane ulnar approach. A residual 3 mL NS was delivered to detach the MN from the FR (Figure 1a to c). In the long-axis group, a total of 5 mL NS was delivered into the intracarpal canal with an in-plane approach to detach the MN from the FR advancing from the wrist crease to the palm (Figure 1d to f).

### Outcome measurements

Another investigator, blinded to the randomization and treatment contents, evaluated all outcome measurements. Assessments were evaluated before injection and at 2 weeks, and 1, 3, and 6 months post-injection. The primary and secondary outcomes were the inter-group differences in mean values of measurements evaluated before injection and at the 6-month follow-up.

### Primary outcome

#### Boston Carpal Tunnel Syndrome Questionnaire (BCTQ)

BCTQ, the most commonly used measurement of CTS symptoms, contains two multi-item scales with a summary score of 0 to 5 for each item; a higher score indicates greater severity. A total of 11 items in the Symptom Severity Scale (SSS) and the 8 questions in the Functional Status Scale (FSS) were used to evaluate the severity of symptoms and functional status, respectively 23. The mean of total SSS and FSS divided by each item score was used for further analysis. The minimal clinically important difference (MCID) was an improvement in SSS score of 0.46 and in FSS score of 0.28 relative to their baseline score (absolute improved SSS or FSS score divided by baseline SSS or FSS = 0.46 or 0.28) 24. The proportion of participants meeting the MCID value was recorded.

### Secondary outcome

#### Cross-sectional area of nerve

The same physician used an electronic caliper to evaluate the cross-sectional area (CSA) of the MN. For reliable results, the patients held their wrists in a neutral position with the palm facing upwards and the fingers in a semi-extended position. The examinations were performed at the proximal inlet of carpal tunnel with the short-axis scan (scaphoid-pisiform level) and where the largest swelling of the MN was identified, as described previously 6, 19. The ultrasonographic evaluation of the CSA of the MN performed at this level has high sensitivity (89%) and specificity (83%) for the diagnosis of CTS 25, 26. Measurements were repeated three times and averaged for further analysis.

#### Electrophysiological study

The same physician performed the examinations to compare the antidromic SNCV and DML of the MN 6, 7, 27. To survey the SNCV, a stimulator was placed 14 cm proximal to the active electrode where the 2^nd^ interphalangeal joint was recorded. To assess the DML, the active electrode was placed on the abductor pollicis brevis with a stimulator at 8 cm proximal to the active electrode. The cutoff values for MN's SNCV and DML for the diagnosis of CTS using electrophysiological assessment were <3.6 ms and <4.3 ms, respectively 20-22. We performed each measurement three times overall and averaged these values for a mean SNCV and DML for statistical analysis 28.

### Sample size

We performed a preliminary power analysis to calculate the sample size (G*power 3.1.9.2, UCLA, Los Angeles, CA, USA) in order to compare the intergroup mean values of BCTQ at baseline and 6 months post-injection.^29^ At least 46 subjects were required to achieve sufficient power ([1-β] = 0.8; α = 0.05; effect size = 0.85 because no preliminary data were available, we used a large effect size of 0.85).

### Data analysis

We used SPSS (IBM Corp. IBM SPSS Statistics for Windows, Version 19.0. Armonk, NY: IBM Corp.) for statistical analysis. A Mann-Whitney U test was used for continuous data and chi-square test/Fisher's exact test was used for categorical data. A Wilcoxon signed-rank and Mann-Whitney U tests were utilized for assessing intra-group and inter-group data at the varying follow-up time points. The 2-way ANOVA was used to test the group by time interaction. Significance was determined as *p*<0.05.

## Results

**Figure 2:**
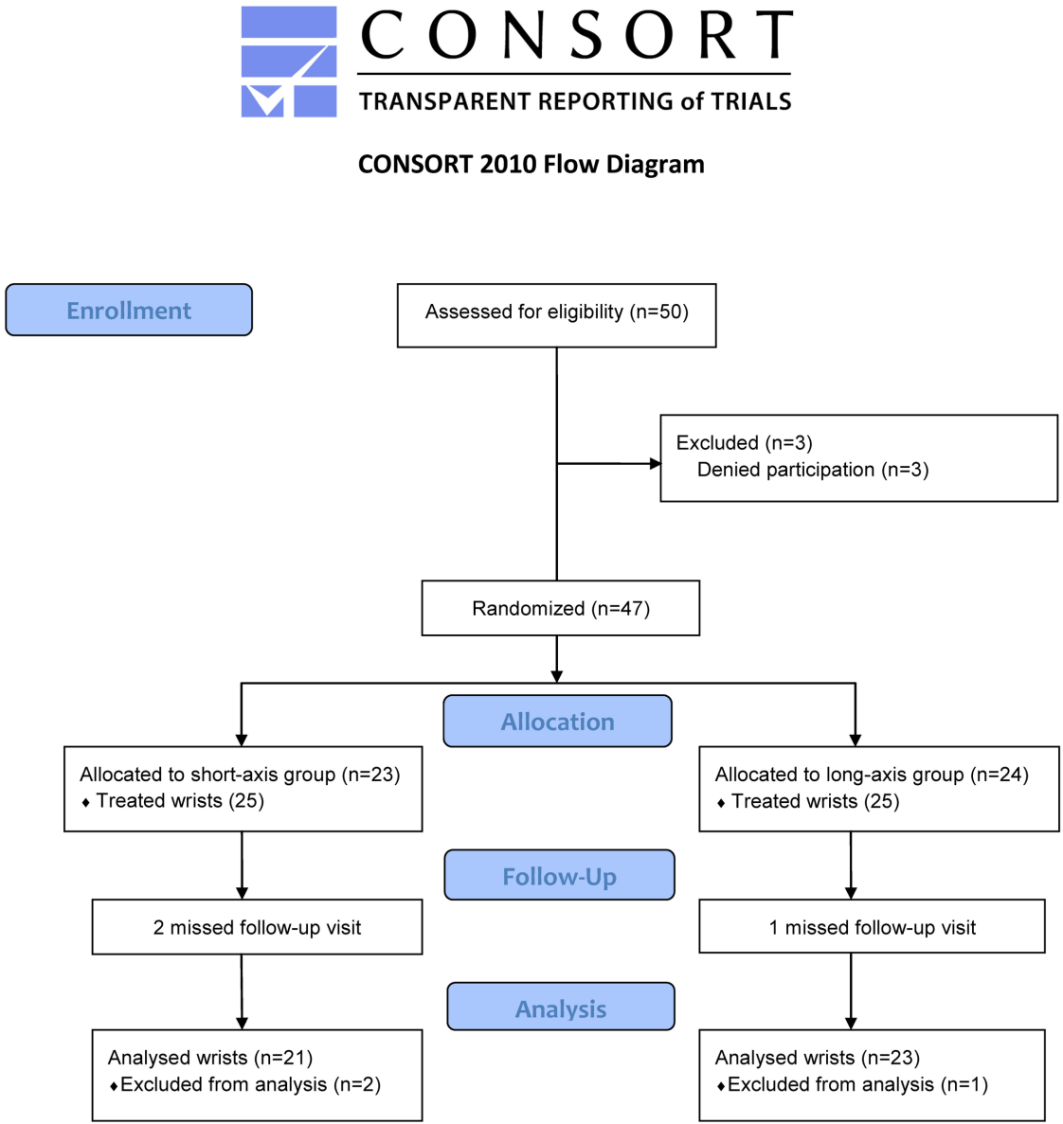
Study flow diagram.

Forty-four participants completed the study (21 wrists in short-axis and 23 wrists in long-axis groups). Two and one patients withdrew from the study due to personal reasons in the short- and long-axis groups, respectively (Figure 2). No statistical differences were found in the demographic and clinical characteristics of the subjects (Table 1).

**Figure 3:**
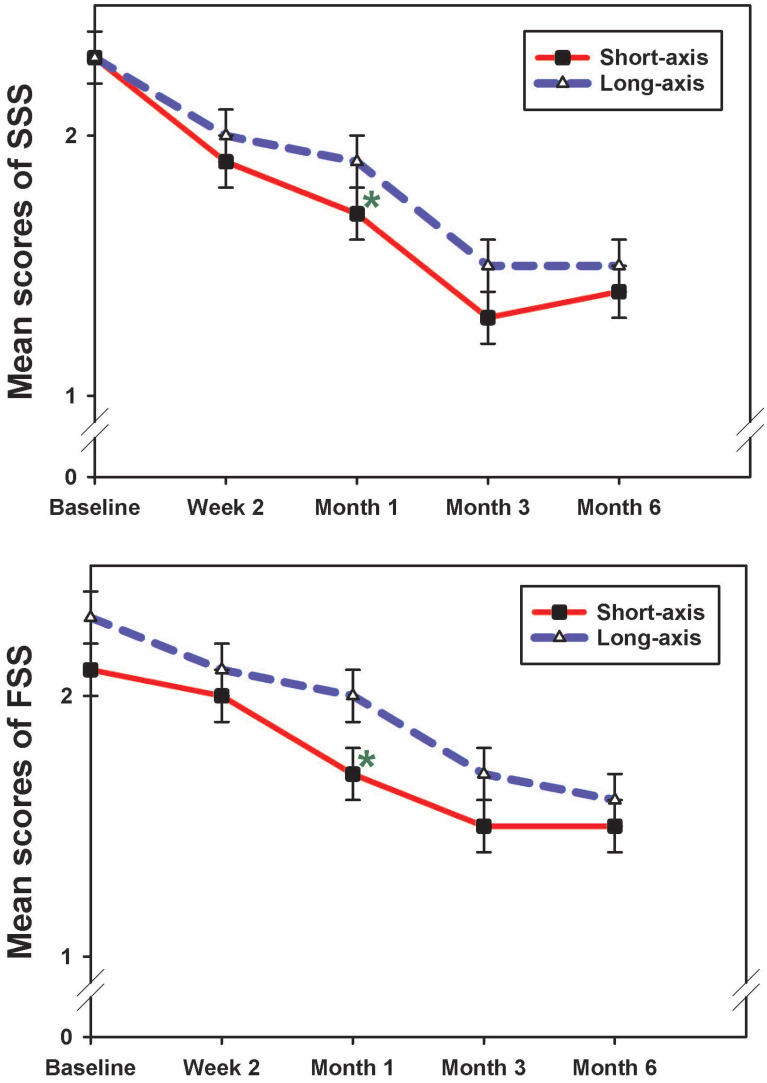
The mean scores of SSS and FSS in both groups at each follow-up time point (mean ± standard error). The result showed a significant reduction of SSS and FSS at 1 month post-injection between groups (short-axis > long-axis group). SSS, symptom severity scale; FSS, functional status scale. *p <0.05; Mann-Whitney U test.

Compared with baseline, both groups showed improved SSS, FSS, and CSA at all follow-up assessments (*p*<0.05) (Table 2). SNCV improved at all follow-up assessments, compared to baseline values in both groups; however, the difference was statistically significant only in the long-axis group (*p*<0.05). The DML result suggested that there was a greater change from baseline at all time points in the long-axis group, which was noticeably, but not significantly, higher at the beginning. However, this change was statistically significant at the 6-month follow-up (p=0.001), while no obvious improvements were observed in the short-axis group (Table 2). A 2-way ANOVA was used to assess whether the overall pattern of change was greater in one group than the other (the group by time interaction). As shown in Table 2, the change was not significant for any of the parameters (p>0.5), except for DML (p=0.049). All measurements did not show significant differences between both groups, except between the 1-month SSS and FSS (short-axis > long-axis group; *p* = 0.031 and 0.023, respectively) (Table 2 and Figure 3). We observed a tendency towards improvement in the electrophysiological study (long-axis > short-axis groups) and CSA (short-axis > long-axis groups) at all follow-up time points (Table 2).

Although the proportion of patients who met the MCID value of the SSS and FSS scores was higher in the short-axis group than in the long-axis group at most follow-up time points, especial for SSS and FSS at the 1-month and 3-month follow-up; however, their intergroup difference was not statistically significant (Table 3). No patients showed obvious complications or adverse effects throughout the study. All patients denied additional medication administration or other treatments throughout the study.

## Discussion

This prospective study found that both short- and long-axis injections were beneficial for mild-to-moderate CTS. Furthermore, short-axis injection was not more effective than long-axis injection, although the short-axis group exhibited a notable reduction in symptoms and disability at 1 month post-injection compared to the long-axis group. Although large improvements in SSS and FSS scores and tendency towards improvement in CSA at most follow-up time points between both groups (short-axis > long-axis group), the difference of BCTQ was not greater than the MCID value and the proportion of patients who met the MCID value of BCTQ between groups; moreover, this difference was not statistically significant. Moreover, the significant improvement from baseline for SNCV and DML was only observed in the long-axis group, and the 2-way ANOVA showed that this difference was significant for DML (p=0.049). Thus, although the improved differences of SSS and FSS between groups were statistically significant at 1-month follow-up, the clinical significance is uncertain. Further studies with a larger sample size are therefore needed to obtain conclusive results.

Studies reported that elevated pressure resulting from an inflamed swollen FR and SSCT could cause MN compression and impaired nerve conduction function 30-32. Even without a substantiated mechanism of HD, the release produced by HD could unleash the trapped nerve and improve gliding resistance. MN remobilization could initiate nerve kinematics rejuvenation, blood flow reperfusion, and nerve re-conduction with the possible downstream effect of nerve regeneration 5-7. Indeed, published research revealed that single HD with 5 mL NS could induce a therapeutic effect for at least 3 to 6 months for mild-to-moderate CTS, which may result from an initial mechanical HD effect with the following possible effect of nerve regeneration 9. In our study, we only recorded the CSA of MN using ultraonography without measuring other parameters such as, enlarged fascicles, echogenicity of the fascicular pattern, or hyperemia using Doppler ultrasound. Therefore, future studies evaluating above ultrasonographic parameters to further understand the mechanism and therapeutic effects of HD are encouraged.

Although various ultrasound guided-injection techniques for CTS have been advancing for decades, earlier studies have shown inconclusive results for their comparative effectiveness. Smith et al. 15 announced that the in-plane short-axis injection combines the benefits of viewing the entire MN and needle presentation with better precision and neurovascular injury prevention. Lee et al. 12 revealed the in-plane short-axis approach above and below MN was better compared to the out-of-plane short-axis approach only above MN. Rayegani et al. 17 demonstrated that the in-plane long-axis approach merely above the MN showed a slightly greater decline in CSA than the in-plane short-axis approach merely below the MN, although no significant intergroup difference was observed. Babaei-Ghazani et al. 16 revealed above or below MN injection were equally effective in reducing symptom/functional scores and improving electrophysiological and sonographic findings.

The possible reasons for the divergence of effectiveness in the aforementioned studies are outlined below. These studies used corticosteroid ± lidocaine injection, which has a strong anti-inflammatory effect and reduces the pressure of the carpal tunnel for symptom relief. The pharmacological effect of the corticosteroid would have a greater impact on the results than the effect of HD, regardless of the method of injection because these studies only used 1-2 mL of injectate, which may have been insufficient to induce HD effect 33. In contrast to the above studies, this study only used NS, so that the mechanical effects of HD alone could be assessed, without any additional pharmacological effects. Hence, the different HD methods in our study are the cause of the different outcomes.

**Figure 4:**
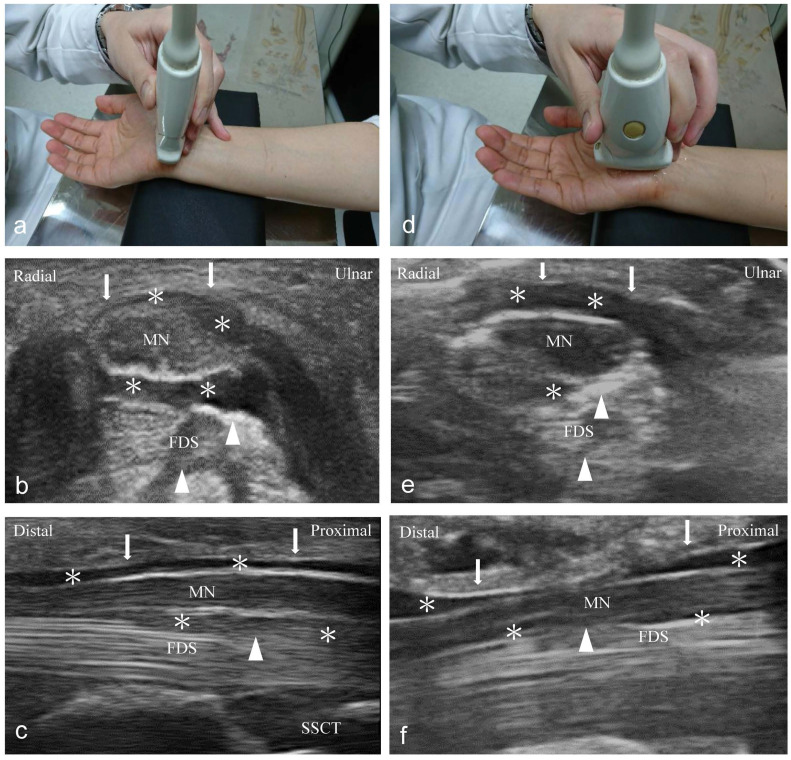
Follow-up ultrasonography imaging after injection (left: short-axis group; right: long-axis group). **(a)** The position of the short-axis scan at the proximal inlet of the carpal tunnel. The injectate (*) can be observed between the median nerve (MN), flexor retinaculum (FR) (arrows), and subsynovial connective tissue (SSCT) (arrowheads) in the short-axis scan **(b)** and long-axis scan **(c)**. **(d)** The position of the long-axis scan from the wrist crease to the palm. The injectate (*) can be observed between the MN, FR (arrows), and SSCT (arrowheads) in short-axis scan **(e)** and long-axis scan **(f)**. Both, the short- and long-axis scans show more injectate (*) between the MN and SSCT in the short-axis group compared to the long-axis group. MN: median nerve; SSCT: subsynovial connective tissue; FDS: flexor digitorum superficialis.

Compared to the short-axis approach, the long-axis injection barely contributes to decreasing adhesion and gliding resistance between the SSCT and MN, although it is supposed to increase the contact area between the FR and the MN via HD. Even though Nwawka et al. 34 showed that the injectate reached 50% and 100% of the MN's circumferential coverage when dissected below and above the MN, respectively, we found more volume distribution between the SSCT and MN (short-axis > long-axis group) (Figure 4). We hypothesize that the intergroup difference might be a result of a greater HD effect between the SSCT and MN, because the adhesion and gliding resistance in these areas contribute to the prominent symptoms of CTS. Although insufficient HD from the proximal-to-distal carpal tunnel via a short-axis injection may be concerning, the following ultrasonography showed complete HD throughout the proximal-to-distal carpal tunnel when using 5 ml of NS (Figure 4c).

The short-duration difference (BCTQ scores at 1 month post-injection) and insignificance of measurements between groups may be due to only a single HD with relatively lower injection volume (5 mL NS) and small sample size. Wu et al. 9 used 5 mL NS single HD similar to our short-axis approach and observed a significant improvement in the SSS on the 2^nd^ and 3^rd^ months and CSA through the 1^st^ to 6^th^ months compared with the placebo group for mild-to-moderate CTS. Compared to Wu's study, both groups of our study received the exact HD with the same injection volume. Higher injection volume and multiple injections could provide more symptom relief with longer persistent effects based on recent studies and clinical observation 35-37. Moreover, the majority of subjects in our study had moderate CTS, and since single HD is typically more effective for mild CTS compared to moderate CTS, this may be another reason. Hence, the short-duration difference between the groups did not extend beyond our prediction. If we use a larger injection volume or multiple injections, we believe that the intergroup difference would extend for a longer duration.

On the contrary, only the long-axis group showed significant improvement of SNCV and DML. These findings could be partially explained based on previous findings that some studies have shown that electrophysiological assessment has limitations in predicting CTS outcomes because routine electrophysiological studies scan chiefly large myelinated fibers instead of small sensory fibers which may contribute to some symptoms of CTS 19, 38-40. However, the trend of increasing improvement (mean difference) over time in the CSA of the MN was observed in the short-axis group compared to the long-axis group, and previous studies reported significant improvement of the CSA of the MN in CTS patients who were satisfied with dextrose injection 7, 28 or surgery 41. Although the relative short-term follow-up may not be sufficient for distinguishing the CSA of the MN between groups in our study, we hypothesize more improvement of mean difference in the CSA between groups would persist if we extend the follow-up peroid. Though only minor therapeutic difference was observed after single HD with 5 mL NS, which may limit its clinical applicability, this study is the first prospective, single-blind, randomized controlled trial to investigate the two different techniques of HD without additional pharmacological effect. Our results make it worthwhile to conduct further well-designed studies using multiple injections or larger injection volume with larger sample sizes and longer follow-up duration to understand the comparative effects of short- and long-axis injections.

Other effectiveness, safety concerns should also be discussed. Previous research advocated that a short-axis scan is superior to a long-axis scan considering that the ultrasound image may be confused as swollen nerve fascicles, muscles, and inflamed tendons in the same plane of the long-axis scan; raising concerns of nerve trauma due to long-axis injection (Figure 1e) 42. Furthermore, the short-axis approach benefits from faster learning with better accuracy of the injection because the operator has better flexible control of the needle from the initial penetration site to the MN which could contribute to injection precision as compared to the long-axis approach (Figure 1) 12, 15, 43. Hence, it may offer a lower risk of nerve injury with a parallel needle approach to the oval-shaped MN that clearly visualizes the whole needle and neurovascular tissue. Nevertheless, the comparison of safety and the learning process between short- and long-axis HD was not performed in our study because all the injections were performed by an experienced physician. As both short- and long-axis HD were effective based on our results, the intervention choice would depend on the operator's preference. We advocate performing an in-plane short-axis intervention above and below the MN, especially for beginners, as this may have the advantage of being safer, easier to learn, and potentially more effective for HD compared to the long-axis approach. Recently, Lam et al. 44 advocated the long-axis approach to the MN from distal to proximal, and start the HD using out-of-plane technique first to release the nerve from the FR and SSCT. When the MN has been released from the soft tissues both above and below, the needle is put back to the top of the nerve and the transducer turns 90 degrees to become in-plane with the needle and the hydrodissecting to more proximal part of the nerve. Although Lam's method can comparatively separate a longer length of nerve via a single insertion point, the learning process is much longer. Further studies are also encouraged to compare the clinical efficacy of the long-axis approach in Lam et al's review and our study.

Our research has a few limitations. First, the actual power of our study (78.6%) was slightly lower than the original calculation (80%) because three patients withdrew from the study, so this may slightly affect the credibility of the results. Hence, further study should assess larger patient population to validate our results. Second, our study does not address the needle placebo effect due to the lack of a sham group; hence, the true effect of HD maybe overestimated. Third, this study did not completely exclude patients with a possible double crush syndrome that could contribute to median neuropathy at the carpal tunnel, and therefore, may have undermined the effect of HD. Fourth; a 6-month follow-up is relatively short and inadequate for comparison with other proven treatment options for CTS. At least a one-year follow-up would be desirable in future research. Final, although no significant intergroup difference was found in the mean value of DML at each time point, the 2-way ANOVA showed that the DML was significantly higher in the long-axis group compared to the short-axis group. Between-group comparison of the mean value may have a larger standard error than comparison of mean change from the baseline; hence, a larger standard error would result in an insignificant difference. Further studies are recommended to compare the mean difference between the groups.

In conclusion, the results of this study suggest that both short- and long-axis HD were effective for patients with mild-to-moderate CTS. The short-axis approach was not more effective than the long-axis injection, although short-axis HD seemed to cause more short-duration improvement of symptoms and disability. Further studies with larger sample sizes, multiple injections, or larger injection volume with long-term follow-up are encouraged in the future.

## Figures and Tables

**Table 1:** Baseline demographic and clinical characteristics of study participants

	Short-axis group (n=21)	Long-axis group (n=23)	^a^*p* value
**Gender, n (%)**			0.449
Female	16 (76.2)	20 (87.0)	
Male	5 (23.8)	3 (13.0)	
Age (year) ± SD (range)	57.7 ± 12.5 (34-78)	59.5 ± 9.9 (32-77)	0.698
BH (cm) ± SD (range)	160.7 ± 5.7 (148-170)	156.9 ± 8.2 (143.5-173)	0.133
BW (kg) ± SD (range)	61.6 ± 7.8 (51-76)	64.3 ± 12.5 (45-87)	0.465
DM (%)	2 (9.5)	5 (21.7)	0.416
Hypertension (%)	11 (52.4)	6 (26.1)	0.074
**Handedness, n (%)**			0.948
Right	20 (95.2)	22 (95.7)	
Left	1 (4.8)	1 (4.3)	
**Lesion site, n (%)**			0.570
Left	10 (47.6)	9 (39.1)	
Right	11 (52.4)	14 (60.9)	
**Padua classification**			0.592
Mild	8 (38.1)	7 (30.4)	
Moderate	13 (61.9)	16 (69.6)	
Duration (month) ± SD (range)	29.9 ± 18.4 (3-60)	22.7 ± 26.2 (3-96)	0.084
SSS (SD)	2.3 ± 0.6	2.3 ± 0.6	0.981
FSS (SD)	2.1 ± 0.3	2.3 ± 0.5	0.228
SNCV (m/s) (SD)	35.0 ± 4.1	33.3 ± 6.4	0.934
DML (ms) (SD)	4.5 ± 0.5	5.1 ± 1.7	0.533
CSA (mm^2^) (SD)	12.4 ± 2.4	12.8 ± 3.7	0.777

BH, Body height; BW, Body weight; DM, Diabetes mellitus; SD, Standard deviation; SSS, Symptom severity scale; FSS, Functional status scale; SNCV, Sensory nerve conduction velocity; DML, Distal motor latency; CSA, Cross-sectional area; ^a^ Mann-Whitney U Test, Chi-square test or Fishers exact test.

**Table 2:** Comparison of changes of BCTQ, electrophysiological study and CSA between both groups

	Short-axis group (n=21)		^a^*P* value	Long-axis group (n=23)		^a^*P* value	^b^*P* value
	Mean ± SE	Mean difference ± SE		Mean ± SE	Mean difference ± SE		
**SSS baseline**	2.3 ± 0.1			2.3 ± 0.1			0.981
Week 2	1.9 ± 0.1	-0.4 ± 0.1	0.001	2.0 ± 0.1	-0.3 ± 1.0	0.004	0.470
Month 1	1.7 ± 0.1	-0.6 ± 0.1	<0.001	1.9 ± 0.1	-0.4 ± 1.1	0.001	0.031
Month 3	1.3 ± 0.1	-1.0 ± 0.1	<0.001	1.5 ± 0.1	-0.8 ± 1.1	<0.001	0.110
Month 6	1.4 ± 0.1	-0.9 ± 0.1	<0.001	1.5 ± 0.1	-0.8 ± 1.5	<0.001	0.776
**FSS baseline**	2.1 ± 0.1			2.3 ± 0.1			0.228
Week 2	2.0 ± 0.1	-0.1 ± 0.1	0.036	2.1 ± 0.1	-0.2 ± 0.1	0.017	0.237
Month 1	1.7 ± 0.1	-0.4 ± 0.1	0.003	2.0 ± 0.1	-0.3 ± 0.1	0.006	0.023
Month 3	1.5 ± 0.1	-0.6 ± 0.1	<0.001	1.7 ± 0.1	-0.6 ± 0.1	<0.001	0.281
Month 6	1.5 ± 0.1	-0.6 ± 0.1	<0.001	1.6 ± 0.1	-0.7 ± 0.1	<0.001	0.476
**SNCV baseline**	35.0 ± 0.9			33.3 ± 1.3			0.934
Month 1	35.6 ± 0.9	0.7 ± 0.3	0.087	34.2 ± 1.3	0.9 ± 0.5	0.035	0.991
Month 3	35.6 ± 1.1	0.7 ± 0.4	0.094	34.6 ± 1.3	1.3 ± 0.5	0.020	0.715
Month 6	36.0 ± 1.1	1.0 ± 0.6	0.120	35.0 ± 1.3	1.7 ± 0.5	0.005	0.787
**DML baseline**	4.5 ± 0.1			5.1 ± 0.4			0.533
Month 1	4.4 ± 0.1	-0.1 ± 0.1	0.452	4.9 ± 0.3	-0.3 ± 0.2	0.124	0.346
Month 3	4.5 ± 0.1	0.0 ± 0.1	0.664	4.8 ± 0.3	-0.3 ± 0.2	0.067	0.805
Month 6	4.4 ± 0.1	-0.1 ± 0.1	0.571	4.6 ± 0.3	-0.5 ± 0.1	0.001	0.981
**CSA baseline**	12.4 ± 0.5			12.8 ± 0.8			0.777
Month 1	11.1 ± 0.5	-1.4 ± 0.2	<0.001	11.6 ± 0.8	-1.2 ± 0.4	0.005	0.869
Month 3	10.6 ± 0.5	-1.8 ± 0.2	<0.001	11.4 ± 0.8	-1.4 ± 0.3	0.001	0.869
Month 6	10.3 ± 0.4	-2.1 ± 0.3	<0.001	11.2 ± 0.7	-1.6 ± 0.4	0.001	0.517

SSS: Symptom severity scale; FSS: Functional status scale; SNCV (m/s): Sensory nerve conduction velocity; DML (ms): Distal motor latency; CSA (mm^2^): Cross-sectional area; SE: Standard error. ^a^Wilcoxon Signed Ranks Test (comparison with baseline, intragroup), ^b^Mann-Whitney U Test (mean, intergroup). The 2-way ANOVA was used to test the group by time interaction, the p-value was 0.706, 0.684, 0.709, 0.049 and 0.537 for SSS, FSS, SNCV, DML and CSA, respectively.

**Table 3:** Proportion of patients meeting MCID of BCTQ between groups

	Short-axis group (n=21)	Long-axis group (n=23)	*p* value
	n (%)	n (%)	
**SSS**			
Week 2	0 (0)	1 (4.3)	>0.99
Month 1	3 (14.3)	1 (4.3)	0.335
Month 3	9 (42.9)	4 (17.4)	0.099
Month 6	4 (19.0)	6 (26.1)	0.724
**FSS**			
Week 2	1 (4.8)	2 (8.7)	>0.99
Month 1	4 (19.0)	4 (17.4)	>0.99
Month 3	16 (76.2)	11 (47.8)	0.054
Month 6	12 (57.1)	12 (52.2)	0.741

MCID, minimal clinically important difference; SSS, Symptom severity scale; FSS, Functional status scale; *p* value from chi-square test or Fisher's exact test.
